# Moving through Toronto’s PATH: Assembling private urban governance

**DOI:** 10.1177/00420980241235371

**Published:** 2024-04-19

**Authors:** Debra Mackinnon, Stefan Treffers, Randy K Lippert

**Affiliations:** Lakehead University, Canada; York University, Canada; University of Windsor, Canada

**Keywords:** assemblage, business improvement areas, condominiums, private urban governance, underground, wayfinding, 集合, 商业改善区, 公寓, 私人城市治理, 地下, 寻路

## Abstract

This paper explores Toronto’s urban PATH, a 30 km network of underground pedestrian tunnels and elevated walkways that connect shopping areas, residential towers, mass transit and downtown destinations. Both as a case and heuristic, this paper situates Toronto’s PATH as an assemblage of private urban governance forms, exploring emergent and evolving constellations of power and responsibility for governing city space that defy easy distinctions of ‘public’ or ‘private’. As an urban assemblage, the PATH comprises potential and actual entities and associations, and is an accumulation of encounters. Never a stable or static entity, the PATH and its governance, we argue, is provisional, revealing constantly evolving connections, alignments and political-economic potentialities. We contend the PATH serves as a palimpsest of mutating governing relations; a multiplicity of meanings, visions and encounters etched into the built environment. By focusing on public and private vestiges, wayfinding, and visibility, and private verticalising ventures, we highlight how practices, logics, processes, urban actors and their histories collide to form fragile, provisional urban alignments and visions.

## 摘要

本文探讨了多伦多市中心的 PATH 地下城系统，这是一个由连接购物区、住宅楼、公共交通和市中心目的地的地下人行隧道和高架走道组成的 30 公里网络。本文针对多伦多的 PATH 进行了案例启发式研究，将其视为私人城市治理形式的集合，探讨了管理城市空间的新兴的和不断演变的权力和责任组合，这些权力和责任组合对“公共”和“私人”的简单区分提出了挑战。作为一个城市集合体， PATH 由潜在的和实际的实体和团体组成，是遭遇的累积。PATH 及其治理从来不是一个稳定或静态的实体，而是临时的，揭示了不断变化的联系、联盟和政治经济潜力。我们认为 PATH 是变异治理关系的体现；大量意义、愿景和遭遇铭刻于建筑环境中。通过关注公共和私人遗迹、寻路和可见性以及私人垂直化冒险，我们强调实践、逻辑、流程、城市参与者及其历史如何相互碰撞，形成脆弱的、临时的城市联盟和愿景。

## Introduction

Beneath Toronto’s downtown streets exists another world, a subterranean network of publicly accessible pedestrian walkways, shopping concourses, attractions and private venues stretching across large swaths of the city’s downtown core. Marketed as the world’s ‘largest underground shopping complex’ and a ‘key mobility network’ (City of Toronto, 2012: 10), the PATH spans more than 30 km and houses over 1200 restaurants, retailers and other businesses and connects several of the Financial District’s major hotels, residential and office buildings, subway stations and Toronto’s tourist and entertainment attractions (City of Toronto, 2023). More a patchwork than network, today, the PATH is almost entirely privately financed, owned, maintained and administered by myriad private governments and public–private partnerships (P3s). The PATH’s governance comprises a complex assemblage of urban actors – multiple major commercial corporations, developers, condominium corporations and business improvement area (BIA) representatives as well as City officials in formal and informal arrangements requiring constant (re)alignment.

This paper situates Toronto’s PATH as an assemblage of private urban governance (PUG) forms, exploring emergent and evolving constellations of power and responsibility for governing city space that defy easy distinctions of ‘public’ or ‘private’. As an assemblage, the PATH comprises potential and actual entities and associations, and like the larger city in which it is situated and its smaller components, is an accumulation of encounters (see Amin and Thrift, 2002; Farías, 2010). Never a stable or static entity, the PATH and its governance, we argue, is provisional, constantly evolving in relation to new connections, alignments and political-economic potentialities. We contend the PATH serves as a ‘palimpsest’^1^ of mutating governing relations, a multiplicity of meanings, visions and encounters etched into the very built environment.

Focused on *vestiges, visibility and ventures*, we highlight how urban practices, logics, processes, actors and their histories collide to form fragile, provisional alignments and *visions*. We show that governance of the PATH is exceedingly complex, involving continual negotiations of maintenance, security and liability responsibilities. Further complicating the PATH is its unruly development and expansion, which for decades has been largely led and financed by individual developers (Bélanger, 2007). The *vestiges* of this dyssynchronous private development have tasked many of its users with navigating a maze-like tunnel system rife with unpredictable twists, turns, dead-ends, disconnections and unfinished spaces. Under the auspices of downtown BIAs and P3s, public and private authorities have periodically sought to render the PATH^2^ more legible, and thus governable through wayfinding and signage efforts to superimpose a cohesive brand and script for consumers and commuters. We argue that *visibility* has become a key governing strategy for the PATH, but one fraught with dilemmas that reflect broader challenges associated with expanding privately governed urban spaces, including aligning disparate and conflicting interests, agendas and *ventures* of the public and private urban agents involved. A venture itself, the PATH comprises and aligns other ventures attempting to protect and enhance various forms of value – whether as property value, rent or commercial revenue. In seeking to explore new connections and encounters within the PATH, the paper also investigates a unique modality of ‘public’ space production and management forged at the intersections of various privately governed realms.

The paper unfolds as follows. First, building on the introduction, we return to prevailing theories of PUG. More than infrastructure or privatisation, we complicate their constitution forwarding a relational and assemblage-based approach to understanding PUG. After a brief description of research methods, we provide an overview of grade-separated pedestrian systems and introduce Toronto’s PATH as both a case study and a heuristic, tracing its early history and illuminating the *vestiges* of interactions among city government and private developers and landowners. Next, we explore how wayfinding efforts to enhance *visibility* in the PATH through the (mis)aligned labours and visions of disparate actors have complicated governance responsibilities. Third, we chart the evolving economic imperatives guiding more recent expansions of the PATH amidst remarkably rapid residential re-development and renewed importance of the PATH in the city’s entrepreneurial aspirations. Specifically, we examine emergent forms of spatial governance and space production at the junctions between heterogeneous PUG forms facilitated by provisional alignments between the city government and condo developers. Concluding the paper, we highlight the indeterminacy and frailty of PUG arrangements in Toronto’s PATH system. We argue that as economic downturns and the recent COVID-19 pandemic demonstrate, the PATH’s assumed economic value as a commercial venture and key mobility infrastructure rests on shaky foundations, requiring (re)*visioning*. We tease out the implications of the PATH for understanding PUG more broadly.

## Assembling PUG: Studying through ‘private’ space and influence

Cities have emerged as strategic assemblages of path-dependent and granular interactions, intersecting with inherited regulatory landscapes and market-orientated restructuring projects (Brenner and Theodore, 2002; Sassen, 2004). The role of public governments has shifted, producing new forms of city space administered by a range of privatised and hybrid governance arrangements. As noted in the introduction, BIAs, condominium complexes, gated communities, retail shopping complexes, privately owned public space (POPS) and special purpose districts have become increasingly common fixtures of an administratively and spatially fragmented urban landscape (Graham and Marvin, 2001; Murray, 2017). The purview of no single authority, these forms represent the more visible and institutionalised manifestations of private power over urban life, mediated through quasi-government institutions, public–private partnerships (P3s) and ‘shadow governments’, further obscuring the costs of their development (Judd et al., 2021; MacLeod, 2011). Often cast (too) broadly as privatisation, these PUG forms feature in discussions of mass private property (Shearing and Stenning, 1981; Zhang, 2017), entrepreneurial urbanism (Harvey, 1989; Ward, 2003), communal space (Kempa et al., 2004), urban gating and carceral walls (Caldeira, 2000), splintering urbanism (Graham and Marvin, 2001), collective consumption (Webster and Glasze, 2006), common interest development (McKenzie, 1994) and mobile urbanism (McCann and Ward, 2011; Peyroux et al., 2012). With notable shifts (see McCann, 2017), these forms and features of urban governance have been discussed from a range of approaches including public administration, community power, political economy, post-political and feminist geographies. Contrary to these theorisations, this paper forwards the utility of urban assemblages and relational approaches for analysing PUG.

Rather than entirely autonomous or self-contained, forms of PUG and the spaces under their remit are entangled in peculiar, ambivalent and often hidden ways. This includes symbolic and immaterial connections such as overlapping governance boundaries and more physical ones like shared infrastructure. Representing more than partnerships, infrastructure or hybridity, PUG highlights the myriad of socio-material processes that co-constitute and co-fabricate urban life. Relational approaches and socio-material turns have sparked much debate^3^ in urban studies – and advanced discussions of flatness, fluidity, scale, structuralism and the complexity of urban processes and the roles of nonhuman agents. *Urban assemblages*^4^ have been proposed as a means of challenging structural meta-narratives of urban life and space to better account for multiplicity, instability, processualism, asymmetry and relationality (Farías, 2010; McFarlane, 2011a; Ong, 2007). With no singular agreed upon history or definition, urban assemblage has been deployed or adopted as a concept, imaginary, analytic tool, descriptive lens and orientation within and beyond urban studies (McFarlane, 2011b; Müller, 2015). As a concept, urban assemblage refers to ‘the processes of construction by which cities, urban phenomena and urban life are constituted’ (Farías, 2011: 369). It offers a way of clarifying how the city is brought into being and is co-constituted by heterogeneous actors, materials and relations, while also recognising the instability, indeterminacy and multiplicity of this process (Farías, 2010; McCann and Ward, 2011). The utility of assemblage ‘is that it describes an entity that has both consistency and fuzzy borders … [it] has some coherence in what it says and what it does, but it continually dissolves and morphs into something new’ (Tampio, 2009: 394). Neither stable nor singular, as Ong (2007: 5) contends, ‘the space of assemblage is the space of neoliberal interventions as well as its resolution of problems of governing and living’.

We discuss assemblage and broader conceptual vocabulary (explicated in context) to explore the productive congruencies of constitution, composition and governance. Governance here is defined broadly to mean ‘any attempt to control or manage any known object’ (Hunt and Wickham, 1994: 78; see also Lippert and Steckle, 2016), including laws, policies, practices and technologies of public and private actors. Governance entails the development of ways to achieve and maintain prescribed objectives (Hunt and Wickham, 1994; Valverde, 2012). Yet this ‘achievement’, much like the PATH, is one in the making, often incomplete, contingent, and subject to shifting objectives and visions. Following the PATH, we trace practices of governance – vestiges, visibilities, ventures and visions – highlighting the relational, productive, heterogenous, territorialising, desired and corporal (see Müller, 2015) features of PUG that (re)make private urban realms.

Echoing this relational approach, and tenants of urban assemblage theories (see also McCann and Ward, 2012), this paper extends research findings from three projects concerning BIAs and condominiums conducted between 2010 and 2022 in Toronto (Lippert, 2010, 2012, 2016, 2019; Mackinnon, 2019, 2021; Treffers and Lippert, 2022). These findings had collectively revealed the PATH connected these and other private sites, and in doing so became a heuristic for thinking through the alignments and entanglements of these intersecting PUG forms. However, to update and narrow this case, data collection and analysis included a range of qualitative methods. Attuned to governing practices, we collected and conducted a content analysis of relevant City, BIA and condominium corporation documents, advertising (*n* = 58) and news media (*n* = 22). We conducted interviews with City employees, BIA staff, condominium board members, and other key stakeholders^5^ (*n* = 10) as well as participant observation in the PATH system between 2017 and 2023. The latter ethnographic components included work-shadowing (see Mac Giollabhuí et al., 2016) City and BIA staff in or linked to the PATH as they carried out daily work (*n* = six visits in 2017 and 2022), allowing us to ‘follow the actors’ (see Cook, 2004; Farías, 2011) and trace the circulation and alignments of governing knowledges and practices in the PATH. Finally, we partook in extensive walking (see Middleton, 2010) of the PATH in the various capacities of consumer, resident, tourist and researcher. Detailed jottings, field notes and photographic diaries were compiled, coded and analysed as a team and informed follow-up interviews. The resulting key themes of vestiges, visibility, ventures and visioning are explored in what follows.

## Private and public vestiges: PATH as palimpsest

Grade-separated pedestrian systems^6^ have been constructed in numerous North American downtowns,^7^ including Minneapolis, St. Paul, De Moines, Dallas, Charlotte, Calgary and Montreal (Boddy, 1992; Maitland, 1992; Yoos and James, 2016a). Contending with suburbanisation and especially malls, grade-separated pedestrian systems were born out of desires for increased density, expansion of valuable city space and ‘interiorisation’ to provide refuge from harsh climates (Boddy, 1992; Maitland, 1992; Robertson, 1987). The emergence of such pedestrian pathways highlights logics of urban pragmatism, tactical urbanism and modernists’ conceptions of efficient ordering of urban space (Yoos and James, 2016a, 2016b). Their size and scale range from isolated skywalks and tunnels to sprawling ‘analogous’ cities (Boddy, 1992) that double and triple the ground plane (Yoos and James, 2016b), exemplifying the volumetric qualities^8^ of the stacked urban fabric (see Graham, 2016; Harris, 2015; Ruming et al., 2021). At the same time, underground pedestrian systems have increased spatial complexity and ambiguity, especially when expanding piecemeal, rather than due to cohesive master planning and design (Boddy, 1992; Yoos and James, 2016b). When lacking vertical and above-grade connections or sufficient spatial cues to orient users, interior urban layers can resemble independent, separate, and untenable realms (Maitland, 1992; Yoos and James, 2016a).

Governance of these pathways has become a complex question (Hopkins, 1996), with many comprising forms of ownership and access that are neither purely ‘public’ nor ‘private’, as well as often shared and continually negotiated management and service responsibilities. Rather than a discrete entity, these pathways contain, abut, and connect a mix of BIAs, condominiums, retailers, offices, government buildings, hotels, tourist attractions and transportation hubs managed and administered by varied actors and organisations. As detailed in the issue introduction, variously conceived as ‘hybrids’ (see Nissen, 2008), POPS (Németh, 2009) or ‘mass private property’ (see Shearing and Stenning, 1981; Zhang, 2017), these pathways highlight the relational character and entanglements among PUG forms.

The history of Toronto’s PATH system and its governance can be traced back to the 1960s when the city was undergoing significant growth and expansion. While the initial underground tunnel connecting the Eaton’s and Simpson’s department stores was already established in 1900 (see Barker, 1986; Fulford, 1995 for a comprehensive history), it was with the development of the financial district and major commercial high-rises in the early 1970s that the ‘vision’ for a comprehensive underground network crystalised (former City Employee). Influenced by central planning ideologies that sought to ‘separate people from traffic’, city planners were keen to expand the underground pathway and provided large subsidies and incentives for developers and property owners to construct below-grade shopping concourses and links (Bélanger, 2007: 276; Stewart, 2016). However, by the mid-1970s with changing city plans and review processes, the attention of city planners turned back towards the ‘street level’, marking a departure of city planning and investment from the underground (Barker, 1986; Stewart, 2016) and leaving much new development to the whims of individual private developers. With unprecedented growth of Toronto’s financial district, expansion of the PATH rapidly accelerated in the 1980s as commercial towers filled in the city’s skyline. Bélanger (2007: 286) characterises this subsequent haphazard and unchecked PATH expansion as driven by the ‘single-mindedness’ of individual developers and property owners ‘seeking opportunistic linkages to connect with other underground nodes’. Indeed, the public sector and civic authorities increasingly considered the underground city to be primarily the private sector’s domain (Morgan, 1997), having replaced development subsidies and incentives with ‘expectations’ of developers and landowners to operate and maintain publicly accessible tunnels, walkways, escalators, lobbies, atriums and shopping concourses (former City Employee; Stewart, 2016). Reflecting the more muted role of city government in the PATH, the goal of encouraging public access remained a general vision of the city after the 1980s and led to proliferation of intersecting POPS managed under a host of agreements and rules concerning maintenance and use (former City Employee). Seen as disparate points of public entry into various commercial properties, governance of the underground was interpolated, sporadic, piecemeal and commonly comprised one-off arrangements between public and private entities. In the late 1980s, a city council endorsed feasibility study recommended ‘a new corporate entity responsible for the underground’ (Morgan, 1997: 3). Initially rejected by the private sector, such an entity would take decades to establish.

The economic downturn in the 1990s interrupted the rapid pace of growth and resulted in the ‘loss of the business association’ and project cancellations that left critical spatial voids as well as chaotic and unpredictable twists, turns and dead ends that are still discernible in the PATH today. Due to absent private leadership, ‘the downtown Business Association kind of fell apart and there was no … private sector downtown voice at the time’, according to one former City Employee. Ambivalence towards the PATH’s development and governance persisted for decades. Despite frequent claims of value (e.g. jobs, property, tax and other revenue generation), public and private visioning and various place-branding efforts (see City of Toronto, 2023; N. Barry Lyons Consultants Limited (NBLC), 2016), neither the City, nor any single private entity, has desired a leading role in PATH governance (see also Stewart, 2016). Rather through its ‘potential’ and practices of (re)inscription, we argue the PATH as an entrepreneurial *venture* has required on-going enrolment of users and expansion of use cases suited to new and shifting economic and policy realities and potentialities.

New partnerships over the past decades highlight the City government’s at-a-distance approach, including tunnel agreements that sometimes have taken nearly a decade to negotiate (see City of Toronto, 2018). For instance, securing the PATH has long been considered by the City and Toronto Police Services as primarily the responsibility of private property owners who contract with a host of private security firms coordinated and overseen by the Financial District Security Group. Primarily concerned with ‘creating clean and safe passage’ (Lippert, 2012) for consumer-commuters, the POPS are heavily surveilled spaces. These are undergirded by extensive, yet independent private security infrastructures that include technologies (e.g. CCTV, PATH COM, automated doors/entrances), personnel (e.g. foot patrols, ambassadors, concierges) and specialised spaces, such as private security offices and holding cells to detain alleged trespassers and thieves until police arrive. Reinforcing existing critiques of POPS (see Németh, 2009), private security practices and infrastructures present challenges to civil liberties and the ‘publicness’ of urban spaces since they facilitate inclusion of prospective consumers and exclusion of those deemed undesirable.

Novel legal agreements also highlight the complexities of public space governance in Toronto’s downtown where responsibilities for maintaining land, including publicly owned land, are increasingly taken up by private landowners. For example, the City has sporadically entered partnerships with private landowners to maintain parcels of City-owned space in exchange for certain benefits. As a BIA Operations Manager revealed:

Where the PATH goes under the street … the City has signed maintenance agreements with the adjacent properties to say, ‘this section of PATH is just to the north of your building, so if you will agree to maintain it, we will allow you to have advertising and you can take that revenue’.

We argue that, as a palimpsest of public and private *vestiges* and relations of history, the PATH can be read as an accumulation of socio-material interactions inscribed into the land, walls, tunnels, retail concourses, incomplete or missing connections and dead-ends. These inscriptions^9^ to some degree continue to script associations and relations (e.g. publicness and openness) as seen with tunnel agreements and private security partnership. Paying attention to ‘practices of gathering, composition, alignment and reuse’ (McFarlane, 2011a: 649), the following sections trace governance through and of the PATH focused on wayfinding and *visibility*, expanding *ventures* and future *visioning*.

## Rendering the PATH visible: Wayfinding partnerships

A common shared experience by those working and visiting downtown Toronto (Daubs, 2023; Fulford, 1995; Morgan, 1997) – from criss-crossing routes to piecemeal directories – the PATH’s illegibility has been a point of public and private intervention. To make sense of decades of haphazard and uncoordinated private development, wayfinding has become the central mandate for the PATH and exemplifies urban governance in which various actors seek to make its pathways more visible, understandable and accessible for users.

In the late 1980s, the City initiated a unified underground wayfinding system led by the Economic Development/BIA office. After nearly a decade of pursuing a reluctant private sector, the City agreed to hire a project coordinator to design a signage system and install it on public property; and in exchange, corporate participants would finance the required signage (Morgan, 1997: 4). However, this implementation faced scepticism from adjoining businesses, and the private sector subsequently requested a coordinated City-led approach that included formal agreements with each property owner, registered on title, ensuring everyone’s commitment and protecting their individual investments (former City Employee). These legal agreements, negotiated by over 30 lawyers, eventually formalised ad-hoc partnerships between the City and property owners and outlined the commitments of stakeholders (see Table 1). Impossible without City funding, this initial wayfinding programme was four times the cost (2 million dollars, split equally by the City and property owners), and took six times longer than estimated with a one to two-year timeline (Morgan, 1997: 8).

**Table 1.** Obligations in the PATH (FDBIA, 2014).

Property owner obligations:	City obligations:
• Purchasing and installing the PATH signs identified on the location plan for the particular building; • Maintaining the identified signs in good condition for 25 years in the future; • Co-operating with the City in installing future map updates and modifying the signage as may be required to reflect new additions to the walkway system	• Purchase, install and maintain PATH signs on City Hall property; • Co-ordinate and monitor the program in the future to ensure that new additions to the walkway system installed PATH signage and that all property owners maintained their signs in good condition; • Initiate map up-date programmes as required; • Investigate ways of enhancing and marketing the PATH logo and other official marks; • Permit the property owners to use the PATH official marks on their signs and in marketing programmes in accordance with the City policy.

Wayfinding efforts were initially deemed successful and rendered the PATH more visible as a whole:

The introduction of PATH signage has successfully increased the identity and visibility of the underground city. The general public have started to refer to the system as ‘PATH’. People notice that the signs are consistent throughout the downtown, and are increasingly perceiving the walkway as public, centrally managed, space. (Morgan, 1997: 7)

However, private development soon outpaced the City’s ability to keep up with legal agreements, and the PATH wayfinding system quickly became outdated. In the 2010s, the search for a new arrangement involving greater private sector leadership resumed (former City Employee). The PATH Masterplan, created by three research consultants in partnership with the City, again identified a lack of leadership and authority. It recommended a PATH Partnership Group (PPG) to oversee the PATH’s redevelopment and governance (see also NBLC, 2016). While the newly established BIA contained most of the PATH in its boundaries, The City BIA Manager noted:

the governance piece was questioned … and they were not interested in taking ownership of this themselves, because they are just one stakeholder in the PATH. That is where the PATH Partnership group concept came up. The idea is that the PATH Partnership Group is meant to be a committee of the BIAs, as well as City divisions, that will … come up with recommendations on all things PATH as they’re needed.

Established in 2013, ‘to address current PATH operational issues and support the future implementation of the PATH Master Plan’, the PPG comprises BIA directors and staff (Financial District, Toronto Downtown West, St. Lawrence Market, Downtown Yonge), City staff across a range of departments (Economic Development and Culture, City Planning, Urban Development Services, and Transportation Services) as well as property groups (FDBIA, 2014). Echoing notions of BIAs as the ‘great convener’, the Operations Manager of the Financial District highlighted the scope and challenges of PATH governance:

Yeah, it is big, and it is a lot. But it is a role BIAs play very well – coordinating between the private and the public sector and making sure everybody is on the same page. It has come a long way from where it was when before the PPG was founded … The reason we don’t have individual private stakeholders involved in the PATH is because the BIAs do a really good job of representing their interests. We basically bear the brunt of advocating for their positions and ensuring that everybody is aware of what their needs are. This is one of the main roles that a BIA plays anyway, it’s just applied in a slightly different way because of … what the PATH is.

Guided by the Master Plan, the PPG focuses on operations and strategic planning (see Table 2) to ensure all stakeholders are aware of guiding documents and to ensure the plan is implemented (FDBIA, 2014).

**Table 2.** PPG areas of focus (FDBIA, 2014).

Property owner obligations:	City obligations:
• Assist in addressing public/private concerns • Be a conduit for information between the public and private sectors • Way-finding • Accessibility • Emergency services access • Tourism • Improving areas where PATH is not up to standard	• Providing support for the adoption of the PATH Master Plan • Providing support to the City for implementation of the PATH Master Plan • Provide support to the City on site-specific issues related to the implementation of the PATH Master Plan, including support of future PATH connections • Improving design of PATH/street level integration • Continue building economic case for value of PATH/ROI for connecting to PATH • Coordinated cultural programming/activation of PATH

**Figure 1.**
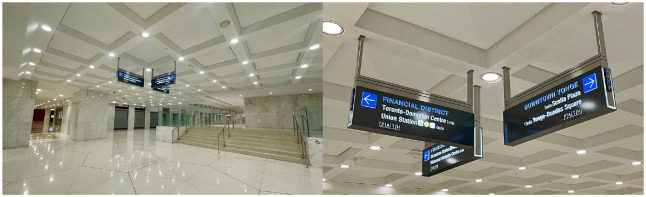
Updated overhead PATH signs and maps. Source: Author’s own, 2024.

Established to update and unify the PATH wayfinding system 2016–2017, the City and the Toronto Financial District Business Improvement Area (TFDBIA)^10^ hired the Steer Group^11^ to create a new brand, graphics, signs and maps (see Figure 1 and City of Toronto, 2020).

We contend this wayfinding evokes forms of ordering that align heterogenous constituents towards the co-production of these brands and boundaries. By ordering space and evoking a regulatory ideal, the PATH brand seeks to shape urban revitalisation trajectories. This governing at-a-distance as well as multiple forms of delegation was discussed by a BIA Operations Manager:

To the user, it looks like one entity is taking care of the PATH. But in reality, First Canadian Place takes care of their section, and TD Centre takes care of their section and Richmond Adelaide Centre takes care of their section and whatever sections of street for the City that they’ve agreed to maintain … The advantage in having the PATH mostly privately owned, and substantially owned by Class AAA Properties, is they take care of it. They all have their own standards of maintenance, their own maintenance staff, they have their own security staff, so it’s taken care of by them.

The PATH’s branding, while creating a unified feel, at once makes PUG visible and masks the actors responsible for it. By scripting movement for consumer-commuters, PATH wayfinding offers a semblance of ‘institutional thickness’^12^ uniting actors towards PUG goals and PPG success. This occurs as municipal services are offloaded in exchange for benefits. However, this cohesive branding, while broadly benefitting constituents, was not universally desired. The PATH, while a conduit and branded network, was not meant to overshadow the branding of specific buildings. An on-going point of re-negotiation, highlighting the limits and on-going constitution of POPS, branding was a repeated concern of properties, as relayed by the BIA Planning and Advocacy Manager:

*It is amazing the things that you do not really understand until you are sitting and talking to the manager of a property, that their concern is that wayfinding for example does not have the same color as a bank logo. That is the level of detail that went into the work behind it.*

Although the designers saw the system as an overlay – wayfinding becomes another corporate layer – this technology required alignment and enrolment of actors towards PUG goals.

The buildings here understand that the PATH map as an example, as a universal asset. When their name changes, they want it changed on every map, not just the map in their building. There is a need to manage mapping and signage from a broader perspective, to ensure it is consistent, correct, and up to date. (BIA Operations Manager)

By delegating governance through what Hermer and Hunt (1996) consider ‘absent experts’, wayfinding also acts as an ‘inscription device’ (Akrich, 1992) describing and attempting to stabilise users of space. While for the BIA, users of the space have predominantly been understood as consumer-commuters – the savvy office workers bypassing traffic, running errands on their breaks, or making the most of office-worker geared amenities – the wayfinding system attempts to enrol other users too, specifically residents and tourists. ‘The PATH has become so expanded and so useful for so many people, it’s not just for businesspeople anymore’ (Koncur, as cited in LeBlanc, 2017). One offshoot of this, however, is the competition of wayfinding and other signage for commuter attention to the point of overload and failure. A research team member reported:

I took the PATH from the CF Eaton Centre back to Union Station. As I exited the somewhat busy mall there were signs covered the glass doors, and I was taken aback by the number of signs in the empty adjoining properties. Far overshadowing the PATH wayfinding, the thoroughfare was full of signboards, roped barriers, posters, vinyl wall coverings alerting users to: new PATH hours, closed routes, as well as public health guidelines. (Field Notes, 2022)

In attempting to create flexible ‘spatial scripts’ (Mackinnon and Richardson, 2017), developer modifications have resulted in inquiries with City and BIA Employees:

One of my neighbours asked the City’s PATH Manager about this diagonal PATH on the map and received the following response: The owner of Maple Leaf Square has requested the City to show the PATH route as a diagonal across the square as their building contains the PATH network at street level and on the 2nd floor. They want the pedestrians to use both of their entrances. The PATH map is meant to be conceptual and not the exact route. (Urban Toronto, 2015)

This failure to provide exact routes, coupled with competing signage, however, caused confusion for another research team member independently walking the PATH (and undoubtedly others). The next tunnel leading to the major attraction, the Eaton Centre shopping centre, had become uncertain, forcing us to approach the lone security guard who became equally bewildered by the PATH map produced, suggesting instead to go up onto Yonge Street level to reach the destination instead (Field Notes, 2022).

These attempts to stabilise users and uses of spaces are ongoing and require intervention to edit the visual mess and render the PATH more legible. For instance, while the Pandemic halted the second rollout of PATH signage, the PPG aims to refresh signage every two years. Yet, this stabilisation hinges on other actors and is intricately entangled with street level developments, as noted in a recent interview with a BIA Planning and Advocacy Manager,

The renaming of Dundas Street will be a particular challenge. It is partially why we held off with rolling out our wayfinding update. A decision was supposed to be made in 2020, then 2021 and now perhaps 2024. We have been trying to align our updates with the renaming, since it will impact the entire system and require changes across the network, which we will coordinate with all buildings.

While wayfinding concerns interpreting environmental cues to locate a destination and involves processes of decision making and information processing (Farr et al., 2012) beyond its locative function, signs that accentuate the City also mediate it (Henkin, 1998). As core constituents of the PPG, to PATH stakeholders wayfinding is not necessarily about getting from A to B. Instead, as noted in the FDBIA’s 2017 strategic plan, it serves broader functions and mandates related to the economic impact and viability of the space, including creating a cohesive brand and scripting user experiences. However, as with the initial stabilisation attempts of the 1990s, these negotiations continue to be thwarted and never fully realised. These dissolving and emergent arrangements and relations highlight shifting constellations of power and decision-making. Not a resultant formation, these ventures require the constant assembling and aligning of heterogeneous actors, agendas, and interests shaped by local contingencies and relational histories.

## Connecting ventures: Condominiums and interstices of private urban realms

Amongst privately governed realms linked to the PATH, the condominium is a relative newcomer. Most PATH-condo linkages have transpired over the last two decades amid rapid proliferation of condo housing in Toronto’s downtown (Lippert, 2019) and increased residential populations commuting via the PATH (City of Toronto, 2012; NBLC, 2016). Currently, over 15 condominiums have direct access to the PATH with others underway. Developers have been eager to market the ‘much sought-after luxury’ and urban connectivity of the PATH. For example, promotional material for a new luxury condominium reads:

[P]eople are looking to invest in communities where everything they need comes with convenience … On cold winter days, you can walk to work, do your banking, visit the doctor and even see a movie without ever having to set one foot outside. This 30-kilometre network … includes … services, restaurants, and endless entertainment; the epitome of convenient urban living. The PATH is truly the heart of Toronto’s financial and entertainment districts … Travelling to your dream tropical destination during the winter months will be easier than ever before. Imagine being able to wear shorts and a t-shirt from home right up until the moment you board your flight without ever putting on your 10-pound winter jacket! With Sugar Wharf’s PATH extension, it will be possible to walk indoors from the waterfront, all the way to Toronto’s world-class Financial District. Equally connected to the city as it is to nature, Sugar Wharf will revolutionize how Torontonians live, work and play. (Sugar Wharf Condominium, 2022)

An attempt to stabilise its potential and value, the PATH is deemed exceedingly beneficial for underground commuting due to its protection of users from Toronto’s harsh winters and crowded and unruly street level sidewalk activity on, for example, Yonge Street. Through corporate storytelling, such conveniences are deployed by private developers to rationalise higher selling prices of units.

**Figure 2.**
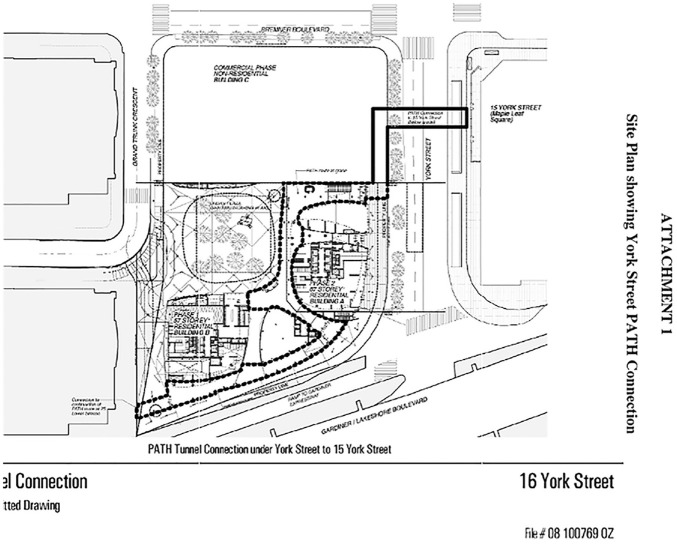
Site plan showing future PATH connections to ICE condominiums. *Source*: City of Toronto (2018).

The rise in PATH linkages over the years stems from legal agreements between City and developers to expand the underground network and enrol more users. Increasingly, the City has required developers to enter Section 16 Agreements^13^ obligating them to finance, construct and maintain connections to the PATH in exchange for the ability to exceed height and density restrictions on their developments^14^ (City of Toronto, 2018). For example, developers of the ICE Condominiums were required to provide, maintain, and operate ‘a publicly accessible PATH walkway … which shall: (1) be fully enclosed and weather protected; (2) remain open and accessible to the public between the hours of 6:00 a.m. and 2:00 a.m., 365 days a year; and (3) be satisfactorily illuminated’ (City of Toronto, 2009: 2). These new spaces would also need to be integrated into the PATH network, with developers obligated to maintain wayfinding signage and construct ‘knock-out’ panels to accommodate potential future PATH connections (see Figure 2). Together, these agreements promise a set of recurring expenses associated with maintaining, repairing, cleaning, and securing new PATH walkways and spaces (totalling 2561 m^2^ in this case) above and beyond original development costs.

However, it is not uncommon for developers to avoid extra financial burdens whenever possible. Concerning ICE Condominium developments, responsibilities for maintaining and operating these new spaces were inscribed in a Complex Reciprocal Agreement by-law created and signed by the developers at the time the buildings were registered in 2016. The by-law transferred the responsibilities to the new condominium corporations that would eventually assume governance of the buildings and the new connections to the PATH, effectively binding future condominium owners to these responsibilities. Though the PATH connection was yet to be constructed, the by-law affirmed that once completed, ‘[t]he costs and expenses incurred in connection with the maintenance, repair and replacement of the Future PATH Connections shall be Common Area Costs’ (Toronto Standard Condominium Corporation [TSCC], 2510: 25). This would also include Wayfinding Agreement Signage: ‘All costs and expenses incurred in connection with the maintenance, repair, and replacement of the Wayfinding Agreement, shall be Common Area Costs’ (TSCC, 2510: 26). The maintenance on its own too should not be assumed to be simple and a minor consideration – Article 7.01 details that ‘Common Areas’ will be,

in good and clean condition and repair and in a safe and sound condition, free of rubbish, debris, ice, snow and other hazards to individuals using the same, such maintenance to include, sweeping and removal of rubbish, trash, garbage and other refuse (the distinction among these remaining unclear), provision of fire protection and security services, adequate heading lighting, ventilating, and air conditioning if appropriate, and maintenance of all necessary electrical and other equipment and facilities. (TSCC, 2510: 26)

These arrangements oblige condominium owners to share collective costs of upkeep and security of PATH connections, funded through monthly common fees, while retaining no ownership stake in them.^15^ And because the provisions are embedded in the condominium’s declaration (often likened to a public government’s constitution), it is exceedingly difficult for owners to collectively opt out of these private governing arrangements. A condominium board member detailed the:

complicated ownership and maintenance structure that is managed in the buildings’ declaration through a bylaw called the complex reciprocal agreement … [that] governs the mechanisms through which we split the costs and who maintains what … And so Cadillac Fairview [neighboring property owner] … actually maintains and operates the public section of our lobbies, including the PATH connection and hallway or tunnel. It is not in the [condominium] corporation’s direct control, but we have influence and we pay for a significant portion of the upkeep for that area because it is a benefit to our residents … we are considered responsible for those costs [for] … operating that tunnel area and making sure it has security and is clean.

These arrangements can then yield governance dilemmas between neighbouring commercial landowners. He further explained that it results in:

a conflict-of-interest situation because obviously they want to minimize their costs. For example, we have this courtyard that is part of the shared lobby area … Cadillac Fairview still maintains it, but really all the benefit of this is to the residents of our building … an office person could use this courtyard, but they never do. They are very slow to repair things …making it not a nice area. That aspect of it does make it hard to have a neighbor like this … Cadillac Fairview is not interested in spending money on a courtyard that does not really benefit their building, but we need them to. It is tricky and it is making it very complicated.

No doubt some condominium residents benefit from links to urban amenities via the PATH, but, vitally, these arrangements reveal the PATH’s governance is not a cohesive private effort of disparate actors working in unison. These are instead ill-fitting private pieces of an assemblage and the everyday practicalities of governing shared private spaces. While some residents mentioned PATH connectivity is commonly prized as a selling point for realtors and landlords, ultimately enhancing property values, the same board member had a gloomier view:

If someone wanted to come from Union Station to our condo, they would have to go upstairs, across the bridge, downstairs, downstairs again, into Longo’s [grocery store], through Longo’s, and then through the tunnel … Who is going to go through that? … they wouldn’t know you would have to go through Longo’s to get to the condominium because logically it doesn’t make sense and there’s not signage to suggest it. Which is why I say, yes technically it is part of the PATH, but I would use that very loosely because no one is going to use it to get to one end of the city to another.

Again, showing the incoherence of wayfinding, PATH connections are cumbersome to navigate, and are consequently underused:

‘I have seen our signage has just been added gradually and very unprofessionally over the years … Very clearly, someone has used Microsoft Word and typed ‘ICE I’ in Times New Roman’ (ICE Board Member).

The arrangements above illustrate a novel form of space production and governance emerging at the interstices of heterogenous private urban realms. While most of the PATH is still owned and managed by commercial landowners, a growing number of new connections are being managed and maintained by condominium corporations and unit owners who collectively fund these narrowly ‘public’ spaces. Rather than revealing a collection of insular, sequestered, or self-contained enclaves (see Murray, 2017) that distribute excludable ‘club goods’ to its private members (Webster, 2002), Toronto’s underground resembles more an accumulation of heterogenous, yet interconnected private realms and ventures, woven together through complex legal agreements, and delimited by less than rigid boundaries of governance responsibilities. The complicated patchwork of privatised governance may elude the typical commuter who traverses these liminal spaces. However, it raises important questions about the eroding publicness of city space as logics of ‘public access’ intersect with a plurality of private interests that seek to imprint their own uses and visions.

## Leaving the path: Vitality and future (re)visions

As with many downtown retail zones, securing the PATH during the COVID-19 pandemic was a formidable challenge. The Financial District Security Group increased patrols and limited points of entry and further scripted movement through the PATH. This lack of effective guardianship has renewed discussions about who bears ultimate responsibility for the PATH and new visioning ventures. One research team member reported:

The once busy stores geared to the now absent commuter-consumers, were less contiguous with every second or third storefront was closed. Some had inventory abandoned on the shelves, some papered up, and many others had prominent and highly branded ‘For Lease’ signs. In a poorly lit section of the PATH beside a closed retailor, one floor to ceiling ad read, ‘I see the PATH to everywhere … I see connected community’. (Field Notes, 2022)

Hard hit by the COVID-19 pandemic, months later the PATH’s future remains uncertain, providing a glimpse into a future devoid of the usual hustle and bustle of downtown employees (see also Daubs, 2023). Not considered a destination itself, the space is vitalised through foot traffic. With food and fashion focused businesses – the sectors most impacted by the pandemic – commercial property realtors predict ‘we’re going to continue to see vacancies down there’ (Saminather, 2021). Media reporting on the PATH since 2020 has focused on short- and long-term recovery. Some remaining businesses have ‘survived’ based on landlord leniency and public subsidies. Reflecting on the loss of businesses in their BIA, one Manager stressed: ‘I think the landlords have done everything they could to keep retailers for as long as they could’. With BIAs broadly advocating across Canada for relief assistance, some recovery mechanisms remain, as the City’s BIA Manager relayed:

COVID-19 recovery has come up a lot… I have councillors and the mayor asking: ‘What are we doing to support businesses in PATH because they were extremely hard hit?’… There’s only so much you could do to bring more business back to the PATH … Thankfully, that’s not my area of responsibility. There is a change that’s happened, an economic shift for the PATH … [it is] going to look a little different. At the very least it is going to take a very, very long time to recover … you’re not going to get so many small retailers back.

Drawing parallels to the 1990s economic downturn that created ‘critical voids’ in the underground system (Bélanger, 2007: 277), public authorities question their role, seeing such matters as market corrections. With economic development officials continuing to claim a withdrawn leadership, the City’s economic injections and broader visioning work nonetheless play a role in sustaining its vitality too. With BIAs in place, the City maintains the PATH is the responsibility of private actors. Yet, within this assemblage, BIAs on behalf of their business members seek to instrumentalise and in a sense govern the City’s funding for their ventures. According to an Operations Manager, BIAs are ‘very focused on promoting the PATH. Before it didn’t need promotion … Now we are really focused … on supporting the new retailers’ and promoting new businesses and events. Through social media campaigns and circulated resources with return to work underway, the five PATH-connected BIAs and property owners hope to re-attract daytime commuter-consumers, enrol new daily active users, and secure ‘more resilient tenants’:

For a long time, the PATH was a lot of the same stuff. There was the person who was working from 9:00 to 5:00 … The retail stores in the PATH existed to serve those people so they could do their shopping, do their dry cleaning, go to the dentist, go the doctor, get their shoes shined … Looking forward, there is a huge opportunity for new things to come in … a slightly different mix of what’s down there … I think we’re going to see a few different uses that try and draw in more of the residents in the downtown core. (Planning and Advocacy Manager)

‘Clothing stores and other traditional businesses that leave could be replaced by educational facilities, clinics, even technology spaces and libraries or other service offerings that are destinations in themselves for residents of downtown Toronto’ (Head of Retail at Oxford Properties, cited in Saminather, 2021).

In considering the longer-term viability of the PATH, its composition is routinely critiqued, with many stakeholders discussing alternatives to make the space more manageable. Limited by City zoning requirements that the downtown core remain offices, private actors, including the BIAs and property managers, are seeking alternatives:

[W]e are looking at … different policies to kind of manage if workers aren’t going to be here as much as they were before. Do we look at more residential development in the area or just what options are available to support our businesses going forward? (BIA Planning Coordinator)

Hoping to chart a new PATH, these private urban actors note the prescriptive and aspirational City vestiges and visions. By chasing the PATH as an economic driver and reinscribing its potentials, developers promise urban community, an anticipatory geography that fails to deliver, where condominium owners, small businesses and others are stuck with the disappointing reality of governing these spaces. Tracing the weaving and co-fabrication of the PATH draws attention to processes and unstable relations that underlie its continuity and change.

## Conclusion

Along with unprecedented growth of high-rise condominiums and other private captures and colonisations of urban skylines in Toronto’s downtown, the underground has become a key frontier for privatisation of the verticalising and volumetric city. Yet, what has been occurring is not only about space and property per se, but also about contingencies of PUG – the actors, emergent practices and materialities, histories and alignments that form urban assemblages and structure the fields of urban possibility.

While the role of PUG (here P3s, BIAs and condominium boards) is to chart new paths through private governance of space – ad-hoc committees, wayfinding, condo agreements and maintenance, ultimately the amassing of vestiges, ventures and visioning continues to thwart the PATH’s cohesion. Viewing the PATH as a palimpsest with decades of (re)inscription, highlights that it is far from a purely private endeavour. Rather, the City – from the tunnel agreements to prescriptive zoning – as well private entities attempt to govern at a distance, highlighting the heterogeneity and public constitution of PUG. While wayfinding and other private PATH ventures seek a cohesive branded space to be governed and enrol new actors, the various actors destabilise institutional thickness. Condominium residents (there is no attached detached residential housing) are repeatedly conceived as a densified and vitalising source by both the City and BIA – yet one, that like the retail ventures, requires security and maintenance. The now hollowed retailed sphere continues to stabilise the PATH as something *in the making*. The concatenation of disparate vestiges, ventures, and vectors creates the conditions of governance as well as gaps and cracks that appear to be filled in by private actors.

The case study of the PATH framed as an assemblage has key implications for understanding contemporary PUG. First, cities are fast becoming a patchwork of privately governed realms, with overlapping and intersecting boundaries, and where divergent public and private interests meet and collide. Rather than sequestered and self-contained enclaves or ‘clubs’, these realms are increasingly intertwined through governing practices, arrangements, and physical infrastructures woven into the interstices of the urban spatial fabric.

Second, the case of the PATH provides no evidence that PUG is a seamless web of private interests conspiratorially working together in unison and coherence. Instead, the governance of the PATH elicits a peculiar incoherence, with ill-fitting private pieces – some far less powerful than and exploited by others, contingently, and historically stitched together to form a frayed quilt spread out over the bowels of the city. In this case, seemingly less coordinated than the ‘revanchist’, ‘carceral’ or ‘punitive’ city, the ambivalence of public and private actors nonetheless furthers administrative fragmentation, delegation, decreased public oversight, and corporate influence.

Third, assemblage and relational approaches help escape and or complicate metanarratives that see the production of space strictly through the lenses of infrastructure, privatisation or neoliberalism (see also McFarlane, 2011a, 2011b). The ontological propositions of urban assemblages offer a means of thinking through spatial formations in the making, including how they are produced, performed, held together, defended, maintained, and repaired (Farías, 2011; McFarlane and Anderson, 2011). By *moving through* we foreground practices and arrangements that seek to render the complexity and chaos of the PATH – its peculiarly arranged spaces, infrastructures and relations – legible and thus governable. But rather than characterising the PATH as entailing ‘co-evolution’ or ‘co-location’ of public and privately supplied goods and spaces, we highlight the messy entanglement, fleeting semblance of thickness, and ongoing negotiations that characterise PUG.

Fourth, relatedly, studying through assemblages reveals processes of underlying continuity and change. The strenuous and fragile process of governing the PATH through the on-going re-inscription of its purpose and value, demonstrates possible implications of this instability. The 30 km PATH in everyday practice is far less than what it purports to or could be. This ambivalence, fragility and frailty opens the possibility for new ways of assembling space more aligned with public use, beyond shallow versions of publicness confined to notions of ‘publicly accessible’ or ‘open’.

## Notes

1. Graham (2013) uses ‘palimpsest’ to theorise the multiplicity of meanings written onto, into, and over space. The concept first referred to mediaeval writing blocks that could be reused while retaining traces of earlier inscriptions: ‘Because those earlier inscriptions could never fully be erased, every writing block was a composite containing trace of all inscriptions’ (Crang, 1998 as cited in Graham, 2013: 117).

2. The ‘PATH’ name is one example of branding exercises undertaken to create a cohesive and recognisable experience for commuters. While not crystalising as vernacular until the late 1990s, we have used PATH throughout to refer to Toronto’s underground pedestrian network.

3. This has resulted in numerous and on-going debates. See, for example, discussions in *City, Dialogues in Human Geography*, (e.g. Acuto, 2011; Anderson et al., 2012; Brenner et al., 2011; Farías, 2011; McFarlane, 2011a, 2011b; Wachsmuth et al., 2011).

4. While basing the concept of urban assemblages on Actor Network Theory (ANT), Farías and Bender (2010) discuss similarities to Deleuze and Guattari ([1980]1987), Callon (1998) and DeLanda’s (2006) use of the term ‘agencements’ and the role of exteriority. Rather than thinking in parts and whole, elements are assumed to be emergent and becoming (Farías, 2010). Others like Müller and Schurr (2016) have updated these debates by highlighting the confluence, slippage, and potential for cross-fertilisation between assemblage and ANT. In considering PUG, we build on these contributions.

5. Most interviews were conducted in 2016 and 2017. Follow-up interviews in 2022 primarily addressed the post-pandemic context.

6. A catchall for various ‘skyways, skywalks, pedways, footbridges, the +15, and the Ville Souteraine’ (Yoos and James, 2016b).

7. By no means limited to North America, Hopkins (1996) and Yoos and James (2016a, 2016b) identify the increasing circulation of such pedestrian systems across Asia and Europe, including in Tokyo, Hong Kong, Beijing, Shanghai, Seoul and Mumbai.

8. Volumetric, implying a particular dimensionality and calculability, is used here to better account for the ‘reach, instability, force, resistance, incline and depth of city development’ (Elden, 2013: 45, see also McNeill, 2020).

9. With key stakeholders providing similar histories, in particular pointing to public and private roles and responsibilities, these accounts serve as scripts (see Akrich, 1992) and attempts at stabilisation. Yet the continued interplay of public and private highlights the messiness of these governing relations.

10. Led primarily by the TFDBIA, this most recent wayfinding system has been championed by City officials and regional, provincial, and international urban place management organisations and has received multiple nominations and awards.

11. This group already had maps and engineering documents to create a distinctive PATH brand consistent with city maps.

12. For Amin and Thrift (1994)‘thickness’ is based on institutional presence, fostering high levels of interactions, a clear mandate, and mutual goal awareness.

13. Section 16 Agreements are similar to a Section 37 Agreements under the Planning Act, both securing community benefits in exchange for height density bonuses (City of Toronto, 2018, see also Biggar and Friendly, 2023; Moore, 2016).

14. See, for example, City of Toronto (n.d).

15. For ICE, ownership of common areas was retained by a neighbouring commercial landowner prior to development.
